# Complement C3 Affects Rac1 Activity in the Developing Brain

**DOI:** 10.3389/fnmol.2018.00150

**Published:** 2018-05-07

**Authors:** Anna Gorelik, Tamar Sapir, Lihi Ben-Reuven, Orly Reiner

**Affiliations:** Department of Molecular Genetics, Weizmann Institute of Science, Rehovot, Israel

**Keywords:** complement C3, cortical development, neuronal stem cells, cell cycle, Rac1

## Abstract

The complement system, which is part of the innate immune response system, has been recently shown to participate in multiple key processes in the developing brain. Here we aimed to elucidate downstream signaling responses linking complement C3, a key molecule of the pathway, to small GTPases, known to affect the cytoskeleton. The expression pattern of the activated small GTPase Rac1 resembled that of complement C3. C3-deficient mice exhibited reduced Rac1 and elevated RhoA activity in comparison with control mice. The most pronounced reduction of Rac1 activity occurred at embryonic day 14. Rac1 has been implicated in neuronal migration as well as neuronal stem cell proliferation and differentiation. Consistent with the reduction in Rac1 activity, the expression of phospho-cofilin, decreased in migrating neurons. Reduced Rac1-GTP was also correlated with a decrease in the expression of progenitor markers (Nestin, Pax6 and Tbr2) and conversely the expression of neuronal markers (Dcx and NeuN) increased in C3 knockout (KO) cortices in comparison with wild-type (WT) cortices. More specifically, C3 deficiency resulted in a reduction in the number of the cells in S-phase and an elevation in the number of cells that precociously exited the cell cycle. Collectively, our findings suggest that C3 impacts the activity of small GTPases resulting in cell cycle defects and premature neuronal differentiation.

## Introduction

Complement acts as a rapid and efficient immune surveillance system that has distinct effects on healthy and altered host cells and foreign intruders (reviews Walport, 2001a,b; Zipfel et al., 2007; Ricklin et al., 2010; Hawksworth et al., 2017). The complement system is composed of a large family of proteins, which are either secreted or membrane bound. These proteins are usually inactive until the system is triggered by stimuli. Complement is activated by three major routes: the classical, the alternative and the lectin pathways, all of which converge on complement component C3, a central molecule in the system that ultimately drives complement effector functions, including the elimination of pathogens, debris and cellular structures. Several complement proteins are cleaved during activation of the system; for example, C3 is cleaved into two fragments, C3a and C3b.

During recent years, the notion that the immune system participates in regulation of complex behavior has emerged and it has been proposed to be malfunctioning in diseases as diverse as autism spectrum disorder (Hsiao et al., 2012; Onore et al., 2012), as well as late onset diseases such as Alzheimer’s (review Veerhuis et al., 2011). Mutations in members of the lectin arm of the complement pathway have been previously implicated in 3MC syndrome (Degn et al., 2011; Rooryck et al., 2011), in which intellectual impairment is part of the complex syndrome. C3 regulates the number and function of glutamatergic synapses in the hippocampus and exerts negative effects on hippocampus-dependent cognitive performance (Perez-Alcazar et al., 2014). C3 deficiency spared age dependent synaptic and neuronal loss in a region-specific manner and protected against cognitive impairment in normal aging of wild-type (WT) mice (Shi et al., 2015). Furthermore, it was shown that C3 deficiency is beneficial in Alzheimer’s disease model as it protected against age- and plaque related synapse and neuron loss, decreased glial reactivity and spared cognitive decline in APP/PS1mice despite an increased plaque burden in the mouse brain (Shi et al., 2017). Innate immune molecules have been found participate in regulation of synaptic plasticity (review Boulanger and Shatz, 2004). The activity of the complement pathway has been implicated in developmental pruning of synapses refinement of the mouse visual system (Stevens et al., 2007; Schafer et al., 2012; Bialas and Stevens, 2013).

Our recent research has shown that immune signaling plays a role in early neural brain development, which includes neuronal stem cell proliferation and neuronal migration (Gorelik et al., 2017a,b). In particular, we have shown that key proteins in the lectin arm of this pathway, MASP1, MASP2 and C3, are expressed in the developing cortex and that neuronal stem cell proliferation and neuronal migration is affected in KO and knockdown mice. Molecular mimics of C3 cleavage products rescued the migration defects that have been seen following knockdown of *C3* or *Masp2*. Pharmacological activation of the downstream receptors rescued *Masp2* and *C3* knockdown as well as *C3* KO (Gorelik et al., 2017a). An additional study investigated the complex developmental roles of *Serping1* or C1 inhibitor, which is known to inhibit the initiation of the complement cascade (Gorelik et al., 2017b). Knockdown or KO of *Serping1* affected neuronal stem cell proliferation and impaired neuronal migration in mice both in a cell-autonomous and non-cell autonomous manner. Most importantly, expression of protein components mimicking cleaved C3 rescued the knockdown of *Serping1*, indicating complement pathway functionality. Despite compelling evidence for the complement functions in the developing brain, the molecular mechanisms that allow these secreted molecules to exert their function is unknown. C3a and C5a receptors are G-protein coupled, which are involved in a wide repertoire of cellular signaling. Activation of G-proteins may induce cytoskeletal rearrangement, which is required for cell division and radial neuronal migration. As key regulators of actin and microtubule cytoskeletons, cell polarity and adhesion, the Rho GTPases play critical roles in CNS neuronal migration (reviews Govek et al., 2011; Evsyukova et al., 2013). Furthermore, activation of the C3a receptor by C3a induced the activity of Rac1, a Rho GTPase, in migrating crest cells (Carmona-Fontaine et al., 2011). Moreover, when Rac1 was inhibited, neural crest cell explants lost their coattraction, supporting the idea that this mechanism occurs by mutual chemoattraction, possibly via Rac1 activated by C3aR upon binding to C3a. Rho family GTPases function as molecular switches and cycle between an active, GTP-bound state, and an inactive, GDP-bound state (review Iden and Collard, 2008). In this current study, we demonstrated the effect of C3 deficiency on the activity of small GTPases, in particular Rac1, and revealed how this affects neuronal stem cell cycle and cell fate determination.

## Materials and Methods

### Antibodies

Mouse anti RAC1 (Millipore, 1:1000), rabbit anti RHOA (Cell Signaling, 1:1000), mouse anti CDC42 (Cytoskeleton, 1:1000), rabbit anti Glutathione-S-Transferase (GST; Santa Cruz, 1:2000) and rabbit anti EMERIN (Santa Cruz, Miami, FL, USA-254 1:1000) were used for western blotting.

The following antibodies were used for immunostainings: mouse anti RAC1 (Millipore, 1:200), mouse anti active RAC1 (NewEast Biosciences, 1:200), rabbit anti C3 (Antibody Verify, 1:400), mouse anti Phospho-COFILIN (Santa Cruz, 1:200), mouse anti iododeoxyuridine (IdU)-B44 (BD Biosciences, 1:200, 347580).

### Animals

Animal protocols were approved by the Weizmann Institute IACUC and were carried out in accordance with their approved guidelines (approval number 34400317-2). *C3* KO mice were obtained from the Jackson Laboratory. Male and female embryos were used in the study.

### Small GTPases Activation Assay

GST-P21 activated kinase (GST-PAK), GST-RHOTEKIN (GST-RTKN) and GST were purified in NETN buffer (0.5% NP-40, 20 mM Tris-HCl pH 8, 100 mM NaCl, 1 mM EDTA) supplemented with PMSF and incubated with glutathione-agarose beads.

Cortices (two brains per tube) from *C3* KO or WT embryos (E16) were collected on ice, immediately dissociated in the lysis buffer (50 mM Tris-HCl pH 7.5; 150 mM NaCl; 1 mM EDTA; 1 mM EGTA; 1% Triton X-100 supplemented with aprotinin, leupeptin, NaF, sodium orthovanadate and protease inhibitor cocktail) and centrifuged at 13,000 rpm. 0.5% of the resulted soup was used directly for western blot analysis to determine the general levels of small GTPases. The rest of the soup from each condition was divided to three equal parts and incubated overnight in 4°C with GST-PAK, GST-RTKN or GST treated beads. The beads were washed three times with NETN buffer. The pelleted beads were eluted by heating in 2× SDS-buffer. The samples were separated by SDS-PAGE and subjected to western blot analysis with the indicated antibodies. The relative quantification of the protein bands was performed with ImageJ “Gels” measurements. The intensity measurements of the bands of the small GTPases from the pull down were normalized to the intensity of the GST bands and to the level of the relevant small GTPase in the brain lysates. Emerin intensity served as the loading control of the lysates. The resulted data from six independent biological repeats of the WT and *C3* KO were analyzed with the *Student* *t*-test. The averages are presented in the graphs.

### IdU/EdU Labeling

The thymidine analogs IdU (0.01 ml of 5 mg/ml IdU solution per gram body weight) and 5-ethynyl-2′-deoxyuridine (EdU, 50 mg per gram body weight) were injected intraperitoneally to pregnant *C3* KO and WT mice (E14) 3 h and 30 min respectively before sacrifice. The brains were removed and fixed in 2.5% PFA-PBS overnight, washed and cryoprotected by immersion in 30% sucrose-PBS solution. The cryosections (10 μm) were pretreated in boiling sodium citrate buffer (10 mM, pH 6) for 30 min. The click reaction was performed with Cy3 azide (2.5 μM) in the PBS-based buffer containing 100 mM Tris-HCl, 1 mM CuSO_4_ and 100 mM ascorbic acid. To block EdU epitopes before immunostaining with anti IdU antibodies the reaction was followed by a click reaction with a non-fluorescent molecule (Phenylthiomethyl-Azide 20 mM, Sigma). After treatment with 10 mM ascorbic acid and 4 mM CuSO_4_, followed by incubation with 20 mM EDTA, the immunostainings with anti-IdU-B44 were performed. The length of S phase was calculated $T_{\text{s-phase}}=\frac{Ti}{L/S},$ given i = labeling interval 2.5 h, L_(leaving)_ = Idu^+^EdU^−^, S_(currently at S-phase)_ = IdU^+^EdU^+^. This protocol was adapted from a previously published procedure (Nowakowski et al., 1989). Overall, click reactions can be successfully combined with immunostainings (Kalveram et al., 2013).

### Immunocytochemistry

Floating vibratome sections (60 μm) or cryosections (10 μm) were permeabilized using 0.1% Triton X-100 and blocked in blocking solution (PBS, 0.1% Triton X-100, 10% HS; 10% FBS) for 60 min. Antibodies were incubated in blocking solution over night at 4°C. After washing, appropriate secondary antibodies (Jackson ImmunoResearch) were diluted in blocking solution, and incubated for 2 h at room temperature. Slices were mounted onto glass slides using Aqua Polymount (Polysciences). Brains of embryos treated with EdU/IdU were fixed in 2.5% PBS-PFA, cryoprotected in 20% sucrose and cryo-sectioned to 14 μm thick slices before further processing.

### Microscopy, Quantification and Statistical Analyses

Images were taken using confocal microscopy (LSM800 Zeiss), equipped with Axio Observer Z1 microscope, and imaged with either Plan-apochromat 20×/0.8, or Plan-apochromat 63×/1.4 oil objectives. The scaling data are 0.624 × 0.624 μm per pixel for 20× magnification, and 0.198 × 0.198 × 0.51 μm per voxol for 60× magnification. The images were processed by ZEN software and/or Imaris software. Cell count and colocalization analyses were performed using Imaris software (Bitplane Inc., Zurich, Switzerland, Imaris core module). Three brains were analyzed for each treatment. Four representative slices from each brain were chosen for analysis. The size of the area of interest was determined and preserved per each experiment. For the cell counts the relevant channel of an area of interest was analyzed with “Spots” module of Imaris, every spot labeling approximate center of the cell body. For double-labeling the new channel was created with Imaris channel mixer and this channel was analyzed with “Spots” module. For the intensity analysis, the mean gray values (ImageJ) of the relevant channel of the identical areas of the cortex were compared. Comparisons of intensities were conducted using the whole width of the imaged cortex. Statistical analysis was performed by *t*-test or *t-test* with Bonferroni correction. Error bars represent standard error.

### Real-Time qRT-PCR

E14 cortices (two brains for each repeat, *N* = 4) from *C3* KO and WT were dissected in cold PBS and RNA isolation was performed according with Sigma protocol (TRI reagent, Sigma). After Dnase treatment (Sigma), first-strand cDNA synthesis was done using M-MLV RT (Promega). Relative levels of expression (three technical repeats for each sample) were normalized to the *29rps* gene. Real-time PCR with SYBR FAST ABI qPCR kit (Kapa Biosystems) was performed using (Quant Studio 5). The following sets of primers were used for Real-time PCR reactions: 29rps: 5′-TCGTTGGGCGTCTGAAGGCAA and 5′-CGGAAGCACTGGCGGCACAT; C3aR: 5′-GGTGAGATGGAGGAACCAGA and 5′-ATTGGGACTGCTAGGCAATG; Nestin: 5′-GCAACTGGCACACCTCAAGA and 5′-AGCAGAGTCCTGTATGTAGCC; Pax6: 5′-CTTTGAGAAGTGTGGGAACCAG and 5′-TGGTTAAAGTCTTCTGCCTGTGAG; Sox2: 5′-TTCGCAGGGAGTTCGCAAAA and 5′-ACCCAGCAAGAACCCTTTCC; Svet1: 5′-GTCGTAGCAACAGGATAGATGAG and 5′-GGCAAACCATTGGGAACTCGTG; NeuroD1: 5′-ACAACAGGAAGTGGAAACATGACC and 5′-CACTCATCTGTCCAGCTTGGG; Tbr2: 5′-GACCTCCAGGGACAATCTGA and 5′-GGCCTACCAAAACACGGATA; Dcx: 5′-GAGTGGGGCTTTCGAGTGAT and 5′-GGAACCACAGCAACTTTTCCAA; NeuN: 5′-GCGGAAACCTCCTCGGACAG and 5′-TTTTCAACGGGTTCAGCGTTCC; Satb2: 5′-CAGCCAGCCAAGTTTCAGAC and 5′-GGAATCATCAAACCTCCCACGG.

## Results

### The Activity of Small GTPases in C3 Brain Lysates

**Figure 1:**
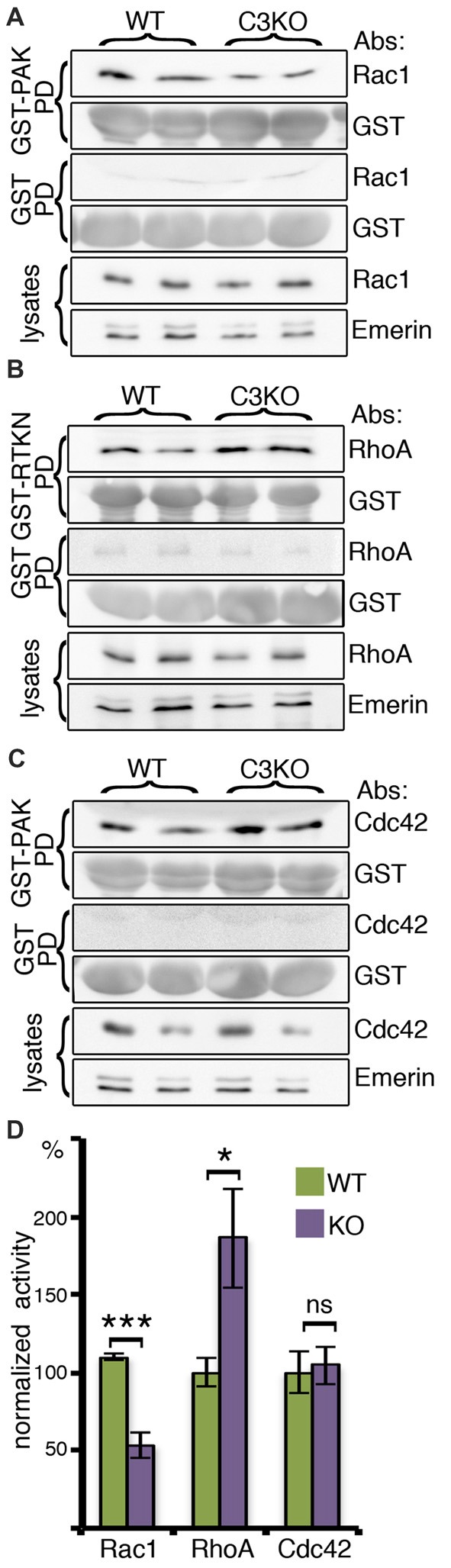
The activity of the small Rho GTPases, RAC1, RHOA and CDC42, in brain lysates. Protein extracts from *C3* knockout (KO; *N* = 6) and wild-type (WT; *N* = 6) E16 cortices were incubated with glutathione-agarose beads pretreated with GST-P21 activated kinase Glutathione-S-Transferase-P21 activated kinase (GST-PAK; **A,C**), GST-Rhotekin Rhotekin (RTKN; **B**) or GST as control for all conditions **(A–C)**. The amount of activated RAC1 **(A)** was determined as a ratio of the amount of RAC1 bound to GST-PAK normalized to the amount of GST-PAK in the reaction and to the total amount of RAC1 in the brain lysate (RAC1/EMERIN in lysates). In a similar way, the activity of RHOA **(B)** and CDC42 was quantified **(C)**. All three proteins did not bind to GST. **(D)** Comparison of the normalized activity is shown. Student’s *t*-test, ***p < 0.001, *p < 0.05, ns, *p* = 0.81.

As mentioned above, previous studies have demonstrated that Rac1 is activated following the binding of C3a to the C3a receptor (Carmona-Fontaine et al., 2011), therefore, we examined the activity of the small GTPases Rac1, RhoA and Cdc42 in the developing brains of WT and *C3* KO mice (Figure 1). The activity of these proteins is regulated by the interaction of Rho family GTPases with guanine-nucleotide exchange factors (GEFs) and GTPase-activating proteins (GAPs). The activated small GTPases bind to their effectors. For example, the GTP-bound Rac1 bind to p21 protein (Cdc42/Rac)-activated kinase 1 (PAK) protein through its Cdc42-Rac-interactive-binding (CRIB) domain (Frost et al., 1997). This property enabled measuring the activity of the small GTPases in brain lysates using pull-down experiments with recombinant proteins expressing the binding domains (from PAK1 for Cdc42 and Rac1 and from Rhotekin (RTKN) for RhoA; Figure 1). The activity was determined by the ratio of the signal of active pulled-down proteins (Rac1, RhoA, or Cdc42) vs. the amount of the relevant recombinant protein in reaction, normalized by the total amount of the small GTPases in the lysate (Figures 1A–C). *C3* KO mice exhibited a significant reduction in Rac1 activity and a significant elevation in RhoA activity, while no change was observed in the activity of Cdc42 (Figure 1D).

### *In Situ* Localization of Rac1-GTP

**Figure 2:**
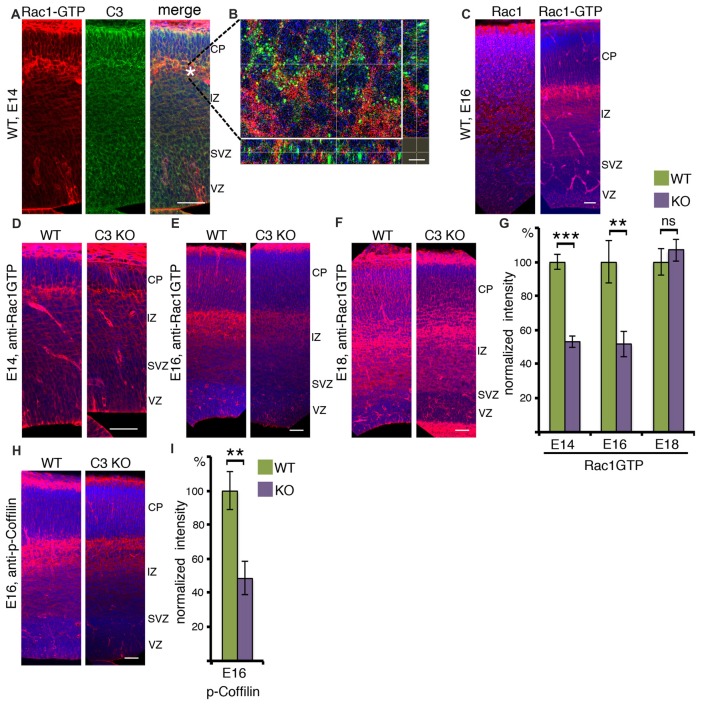
Activated RAC1 in the developing cortex. **(A)** E14 brain sections were immunostained with anti-RAC1-GTP and anti-C3 antibodies. RAC1-GTP shows the highest intensity on the entrance to the cortical plate (CP). The scale bar is 50 μm. **(B)** High-magnification shows that in this area the signal of C3 is in close vicinity to that of RAC1-GTP. The scale bar is 5 μm. **(C)** E16 brain sections were immunostained with anti-RAC1 or anti-RAC1-GTP antibodies. Total RAC1 was evenly distributed all over the cortex, whereas the intensity of RAC1-GTP immunostaining signal was the highest between intermediate zone (IZ) and CP. The scale bar is 50 μm. **(D–F)** Comparison of anti-RAC1-GTP immunostaining in WT and *C3* KO on different embryonic days: E14 (**D**, *N* = 6), E16 (**E**, *N* = 6), E18 (**F**, *N* = 6). **(G)** The normalized intensity of RAC1-GTP is presented as percentage of WT levels. **(H)** WT and *C3* KO E16 brain sections (*N* = 6) were immunostained with anti-phospho-COFILIN antibodies. The scale bars are 50 μm. **(I)** The normalized intensity of phospho-COFILIN is presented as percentage of WT levels. Student’s *t*-test, ***p < 0.001, **p < 0.01.

To better understand how the reduction in Rac1 activity in C3 KO impacts brain development, immunostainings with anti-Rac1 and anti-C3 antibodies were conducted on E14 embryonic brain slices (Figures 2A,B). The distribution of the active Rac1 signal was most pronounced in the area which is in between the intermediate zone and the cortical plate, the subplate, and in the marginal zone. Prior to entering the cortical plate migrating neurons usually change their polarity (Tabata and Nakajima, 2003). Interestingly, immunostainings with anti-C3 antibodies demonstrate an increased signal in the same domain (Figure 2A). Higher magnification images reveal that in many cases the C3 signal is juxtaposed to active Rac1, which could be expected from an extracellular secreted molecule and an intracellular signaling molecule, respectively (Figure 2B). We next proceeded to examine whether the expression of total Rac1 differs from that of active Rac1. At E16, Rac1 is widely expressed in the developing brain and a more pronounced signal can be seen in the intermediate zone and the cortical plate. Active Rac1 strongest signal was observed in the subplate similar to E14 (Figure 2C). Then, we examined how active Rac1 is modulated in embryonic C3 KO brain (Figures 2D–G). At embryonic day 14 and 16, Rac1 activity was markedly and significantly reduced in C3 deficient brain sections (Figures 2D,E,G). Rac1 activity at embryonic day 18 did not differ between the C3 KO and the WT brains (Figures 2F,G). Rac1 has multiple downstream targets, amongst them it is known to induce the phosphorylation of Cofilin, which promotes actin polymerization (Delorme et al., 2007). Furthermore, previous studies have demonstrated that Cofilin is phosphorylated in migrating neurons, downstream to the Reelin pathway, and this activity is important to promote neuronal migration (Chai et al., 2009; Frotscher et al., 2017). Therefore, E16 brain sections were immunostained with anti-phospho-Cofilin antibodies (Figure 2H). The sections from C3 KO mice displayed significantly reduced levels of phospho-Cofilin, which mirrored the reduction in activated Rac1 (Figure 2I). Interestingly, the reduction in Rac1 activity was temporal and correlated well with the developmental peak in neuronal stem cell proliferation and neuronal migration. We speculate that at least part of the neuronal migration impairments observed in the developing C3 deficient mice are due to the reduction in active Rac1 and consequently phosphorylated Cofilin (Gorelik et al., 2017a).

### Rac1-GTP in the VZ and SVZ

**Figure 3:**
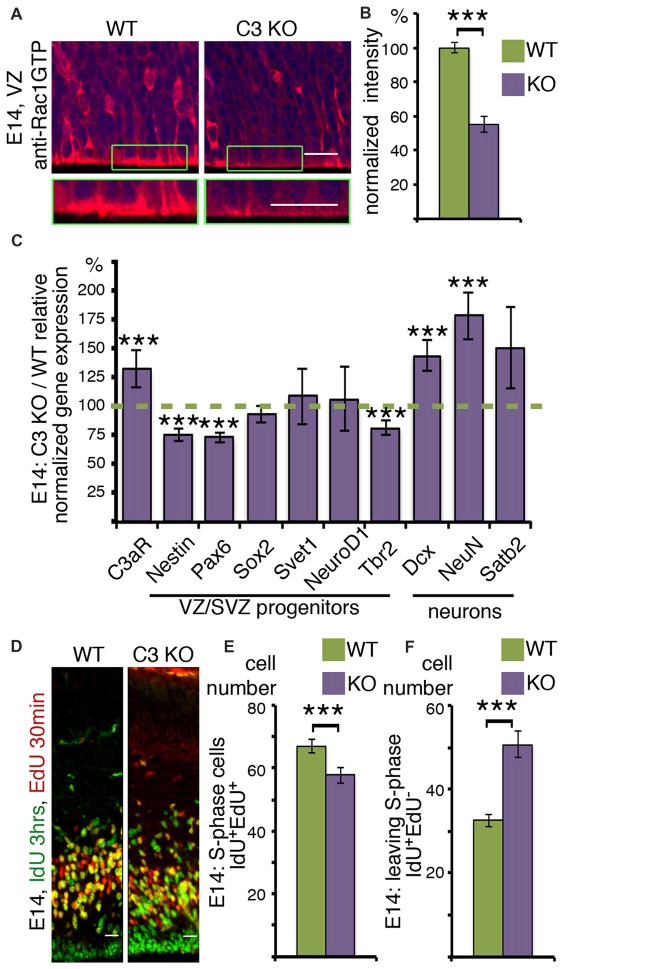
*C3* KO affects neuronal cell fate decisions and stem cell porliferation. **(A,B)** Ventricular zone (VZ) area of E14 WT and C3 KO brain sections (*N* = 6) immunostained with anti-RAC1-GTP antibodies. A higher magnification is shown in the green boxed insert. **(B)** The intensity of RAC1-GTP was measured and shown as percentage of WT levels. Student’s *t*-test, ***p < 0.001 **(C)** Results of real-time qRT-PCR are presented as the ratio of mRNA expression levels of *C3* KO to the WT. Two technical repeats of four brains for each genotype were used for analysis. *Student’s* *t*-test with Bonferroni correction, ***p < 0.001. **(D)** *C3* KO and WT embryonic brains were labeled at E14 with IdU (3 h) and EdU (30 min). The brains were cryosectioned, EdU was detected by Cu(I)-catalyzed [3 + 2] cycloaddition reaction followed by immunostainings with anti-IdU antibodies. The scale bar is 20 μm. The number of cells per equal area which are actively in S-phase (IdU^+^EdU^+^; **E**) and leaving the cell cycle (IdU^+^EdU^−^; **F**) compared between *C3* KO (*N* = 17) and WT (*N* = 22), Student’s *t*-test, ***p < 0.001.

Next, the proliferative zones, which include the ventricular zone (VZ) and subVZ (SVZ) were examined more closely (Figure 3). Rac1-GTP is localized mainly to the apical membrane in the VZ (Figure 3A and insert), with some cells showing a signal in the cell soma (Figure 3A). Rac1 activity is significantly and markedly reduced in the same areas in C3 KO mice (Figures 3A,B). Reduced Rac1 activity within these stem cell niches may affect neuronal cell fate decision as well as cell cycle parameters. Therefore, we examined the steady state mRNA status of a battery of genes using RNA extracted from WT and C3 KO E14 cortices (Figure 3C). The expression of the C3a receptor C3aR, increased in more than 25% in the C3 KO, probably reflecting some compensatory pathways due to the absence of C3 and its cleavage products C3a and C3b. This finding may suggest that in C3 KO there may be alterations in other complement-related genes. Next we proceeded to investigate the expression of a battery of progenitor markers by means of real-time qPCR. A significant reduction was noted in the expression of two progenitor markers, Nestin and Pax6. However, the expression of Sox2, which labels preferentially radial glia, but also intermediate progenitor cells (Hutton and Pevny, 2011) and Svet1, which labels multipolar cells in the SVZ (Tarabykin et al., 2001; Sasaki et al., 2008) did not vary between the WT and the control brains. In a similar manner, the expression of NeuroD1, which is highly expressed in the VZ, is upregulated several fold in the SVZ, and then is downregulated in the CP (Pataskar et al., 2016), was similar between the two genotypes. The expression of the intermediate progenitor marker, Tbr2 (Kowalczyk et al., 2009), was significantly reduced in the C3 KO mice. In a converse fashion, there was a significant increase in the expression of two early neuronal markers, Dcx (Francis et al., 1999) and NeuN (Mullen et al., 1992). Changes in the expression of the neuronal upper layer marker Satb2 (Britanova et al., 2008) were not significant. The observed decrease in some of the progenitor markers and the increase in some of the neuronal markers suggest that in C3 KO there may be a reduction in some of the neuronal progenitors which results in premature differentiation.

To gain a direct insight on cell cycle parameters, the progenitors were double labeled with two thymidine analogs, which are incorporated into the DNA during S-phase. The first label was done with IdU, followed by a second label with EdU. IdU was detected by immunostaining and EdU incorporation was visualized by click chemistry. The nuclei which are double labeled with both analogs either remained in S-phase for the duration of the combined labeling time or re-entered S-phase. The number of double positive cells decreased in the C3 KO mice (Figure 3E). Conversely, the number of cells that exited S-phase and were exclusively labeled by IdU, was higher in the C3 deficient embryos (Figure 3F). The duration of S-phase was 5.24 ± 0.75 h in the WT animals and only 2.96 ± 0.64 h in the C3 KO mice. In summary, C3 KO mice exhibited significant deviations in S-phase duration from the WT mice. Collectively, our study demonstrates that deficiency of C3 affected not only neuronal migration (Gorelik et al., 2017a) but also impaired cell cycle progression and the expression of cell fate markers.

## Discussion

Our current study suggests that the small GTPase Rac1 may be participating in mediating the signaling following complement activation in the developing brain. Small Rho GTPases are known to mediate cytoskeletal changes involving both the actin and the microtubule cytoskeleton, both of which are important for CNS development (reviews Govek et al., 2011; Evsyukova et al., 2013). Our study detected that *C3* KO mice exhibit a significant reduction in Rac1 activity and a significant elevation in RhoA activity, while no change was observed in the activity of Cdc42. The reduction in Rac1 and elevation in RhoA fits with the known activities of these small GTPases. From the perspective of cell morphology, Rac1 and RhoA oppose each other. Canonical descriptions of cell migration place active Rac1 at the front of a migrating cell and active RhoA at its rear. Biochemically, Rac1 and RhoA are generally found to interact in mutually antagonistic ways, playing opposing roles in cell migration (review Guilluy et al., 2011). These opposing activities, as well as a negative feedback loop, lead to bistability in the signals regulating the dynamic cytoskeleton (Byrne et al., 2016). The availability of antibodies which recognize the active, GTP-bound form of Rac1 activation enabled the visualization of the temporal and spatial specific activity in the developing brain. Rac1 activity peaked at E14, coinciding with active neuronal migration and neuroblast proliferation. Migrating neurons entering the cortical plate demonstrated a strong Rac1-GTP signal which was reduced in C3 KO. The documented role of C3 in refinement of synaptic connections (Stevens et al., 2007) and the pattern of active Rac1 localization may point at additional and yet unexplored roles of C3 in early synaptogenesis. We found a particular high Rac1 GTP in the subplate, a transient zone that is located below the cortical plate and above the intermediate zone in the rodent developing cortex. The marginal zone and subplate neurons are among the first to be become post mitotic and they contribute to axon pathfinding and to circuits establishment (Balslev et al., 1996; López-Bendito and Molnár, 2003).

We further investigated the phosphorylation status of Cofilin, which is one of the downstream targets of Rac1. Cofilin is also known to promote neuronal migration and acts downstream to the Reelin pathway (Chai et al., 2009; Frotscher et al., 2017). The phosphorylation of Cofilin in C3 KO brains, was markedly reduced. Rac1 conditional KO mice exhibited delayed neuronal migration of pyramidal neurons (Chen et al., 2007). The phenotype observed in these Rac1 conditional KO mice was similar to that documented in *C3* KO, where neuronal migration was hindered but not completely halted (Gorelik et al., 2017a). Our previous studies have shown that the active lectin/MASP arm of the complement pathway, leading to cleavage of C3, and activating the complement peptide receptors for C3a and C5a, is required for proper migration of neurons in the developing brain (Gorelik et al., 2017a). Furthermore, we suggested that functional activation of the pathway, resulting in C3 cleavage and production of C3 and C5 bioactive mediators, may be an important step in shaping the journey of neurons on their way to the cortical plate. Based on our current findings, we propose that Rac1 is one of the mediators of the complement signaling and that Rac1 facilitates transmission of the signal to the cytoskeleton, which will in turn mobilize the migrating neurons.

The possible effect on Rac1 activity was further investigated in the VZ and SVZ zones. A prominent signal of Rac1-GTP was noticed in the apical domain of neuroblasts in the VZ, which confirms previous studies (Minobe et al., 2009). In that study, addition of a Rac inhibitor or forced expression of a dominant-negative Rac1 significantly retarded interkinetic nuclear migration, and resulted in cytokinesis failures (Minobe et al., 2009). A role for Rac1 in the production of neuronal progenitors and cell fate specification was shown using a conditional allele (Chen et al., 2009). Rac1 deletion in the telencephalic VZ progenitors resulted in reduced sizes of both the striatum and cerebral cortex. Neuronal progenitors exhibited accelerated cell-cycle exit and increased apoptosis during early corticogenesis, leading to a decrease of the neural progenitor pool in mid-to-late telencephalic development (E16.5 to E18.5; Chen et al., 2009). Here, we have demonstrated a significant reduction in the length of S-phase and an increased exit from the cell cycle in the *C3* deficient mice. We propose that complement C3 KO resulted in modified expression of some key progenitor genes. Recent studies demonstrate that at the single cell level there is a huge variability in gene expression levels, therefore, it is possible that the lack of C3 affects different progenitor populations in diverse manners. The expression of *Nestin* was significantly reduced following C3 KO; *Nestin* mRNA expression correlates with many, but not all, regions of proliferating CNS progenitor cells. In addition to its temporal and spatial regulation, *Nestin* expression is also regulated at the level of subcellular mRNA localization: in columnar neuroepithelial and radial glial cells *Nestin* mRNA was predominantly localized to the pial endfeet where most of the active Rac1 signal was localized to (Dahlstrand et al., 1995). In a similar manner, the expression of the key progenitor maker *Pax6* was reduced in C3 KO brains. The levels of *Pax6* are known to be essential for controlling the balance between neural stem cell self-renewal and neurogenesis (Sansom et al., 2009). However, the expression of *Sox2* did not vary. Within the second population of progenitors, the intermediate progenitors, a reduction in the expression of the transcription factor Tbr2 (Kowalczyk et al., 2009), was noted, but no changes were noted in the expression of *Svet1*. One consequence of the reduced expression of some progenitor gene makers, shortening of S-phase and an increased exit from S-phase could be premature differentiation and depletion of the pool of progenitors. Indeed, we noted increased expression of the neuronal markers *Dcx* and *NeuN*, and in our previous studies we detected a reduction in the width of *C3* KO cortices (Gorelik et al., 2017a). Collectively, our findings implicate Rac1 as one of the important downstream mediators of complement activity within the developing brain of the mouse embryo.

## Author Contributions

AG planned, conducted and analyzed most of the experiments. TS and LB-R planned, conducted and analyzed some of the experiments. OR planned and analyzed the experiments. All the authors participated in writing of the manuscript. All authors agree to be accountable for the content of the work.

## Conflict of Interest Statement

The authors declare that the research was conducted in the absence of any commercial or financial relationships that could be construed as a potential conflict of interest.
